# Monophyly or Paraphyly– The Taxonomy of *Holcoglossum* (Aeridinae: Orchidaceae)

**DOI:** 10.1371/journal.pone.0052050

**Published:** 2012-12-14

**Authors:** Xiaoguo Xiang, Dezhu Li, Xiaohua Jin, Hao Hu, Hailang Zhou, Weitao Jin, Yangjun Lai

**Affiliations:** 1 State Key Laboratory of Systematic and Evolutionary Botany, Institute of Botany, Chinese Academy of Sciences, Beijing, China; 2 Key Laboratory of Biodiversity and Biogeography, Kunming Institute of Botany, Chinese Academy of Sciences, Kunming, Yunnan, China; 3 Xi’an Botanical Garden, Xi’an, Shaanxi, China; Biodiversity Insitute of Ontario - University of Guelph, Canada

## Abstract

Recently, there have been a lot of intense debates about the acceptance/rejection of paraphyletic groups in biological classification. On the one hand, evolutionary classification states that similarity and common descent are two criteria for biological classification and paraphyletic groups are natural units of biological classification. On the other hand, cladistic classification considers that common descent is the only criterion in biological classification and monophyly should be strictly adhered to. *Holcoglossum* is used herein as a case to illustrate this problem. Although *Holcoglossum* is a small orchid genus of less than 20 species, there is little consensus about its generic circumscription since it was established, which leads to confusion in taxonomic treatments in the *Aerides-Vanda* group. Based on the analyses of molecular and morphological evidence, our results suggest that the clade comprising *Holcoglossum* s.s., *Ascolabium*, *Penkimia* and *Ascocentrum himalaicum* is strongly supported as a monophyly, and that the three taxa are nested within different subclades of *Holcoglossum* s.s. Thus, it is reasonable to recognize a monophyletic circumscription of *Holcoglossum*, which is also well supported by some vegetative and floral characters. The *Holcoglossum* s.l. would facilitate a better understanding of pollinator-driven floral divergence and vegetative stasis than a paraphyletic and narrowly defined genus.

## Introduction

In the era of integrative taxonomy, there is more major congruence in the biological classification between cladistic classification and evolutionary classification except with regard to the acceptance/rejection of paraphyletic groups. Phylogenetic (cladistic) methods and DNA sequences are routinely used in systematics and taxonomy, and both schools of classification have recognized that the principle of common decent plays a major role in biological classification. However, the fundamental question about the acceptance/rejection of paraphyletic groups remains unsolved, and recently there have been many intense debates about this issue [1]–[11]. Evolutionary classification recognizing paraphyletic groups argues the following: (1) there are two criteria for biological classification, i.e., similarity and common descent [12]; (2) many species are paraphyletic [1], [12]–[14]; (3) paraphyletic groups are natural transitional stages in the evolution of taxa and are natural units of biological classification [4]; (4) cladistic classification is incompatible with the Linnean hierarchy system [1], [4], [7], such as diachronous groups and (5) classification based only on common descent often fails to reflect divergence and natural selection. In contrast, cladistic classification states the following: (1) only monophyletic groups in their strictest sense (holophyly) that are evidenced by synapomorphous characters are recognized in biological classification; (2) only species and clades are objective, and supraspecific taxa are terminals in cladistic classification [15], [16]; (3) there are no objective criteria to circumscribe paraphyletic groups, and paraphyletic groups are artificial classes created by taxonomists to emphasize some particular characters or divergence [5], [16]–[18] and (4) cladistic classification can be accommodated within the Linnaean system except for monotypic higher taxa and historic groups [19], with the former being neither paraphyletic nor monophyletic and the latter being dismissed [16], [19]. However, as there are many theorical and lengthy discussions anywhere [1]–[11], a discussion of the merits and fallacies of each school of classification is not the major aim of present paper. Instead, we used the taxonomy of *Holcoglossum* (Aeridinae, Orchidaceae) as a case to illustrate this problem here.

The orchid subtribe Aeridinae is a large and well-defined horticulturally important group of approximately 1200 species in 120 genera [20], [21]. However, the taxonomy of Aeridinae, particularly the generic delimitations, is difficult and has been considered as “the black pit” [20]–[26]. Seidenfaden [27] (page 8) even stated, “A recurrent dilemma in the study of the Aeridinae is the allotment of a species or a group of species to a genus. − I have several times met with this problem, e.g., in *Aerides*, *Holcoglossum* and *Ascocentrum*.”

*Holcoglossum* is a small Asian genus, consisting of less than 20 species and mainly distributed in southwestern China and neighboring regions. Nonetheless, *Holcoglossum* occupies an important systematic position in the informal taxonomic group, the *Aerides*-*Vanda* alliance, which includes *Aerides*, *Ascocentrum*, *Ascolabium*, *Holcoglossum*, *Neofinetia*, *Papilionanthe*, *Penkimia*, *Seidenfadenia* and *Vanda*. *Holcoglossum* has been redefined several times since it was established by Schlechter [27], but it appears that there is little consensus about its generic circumscription, with many species being transferred among genera within the *Aerides-Vanda* alliance [22]–[24], [28]–[33]. Based on plastid *matK* and *trnL*-*F* and nuclear ITS sequences, two most recent molecular phylogenetic studies of *Holcoglossum* [32], [33] have helped to clarify this problem; however, their results of each are substantially different, thus further confusing the taxonomy of *Vanda-Aeridies* alliance [32], [33].

In the present study, phylogenetic relationships were inferred using four DNA markers (plastid *matK*, *trnH*-*psbA* and *trnL*-*F*, and nuclear ITS sequences) and combined with morphological analyses with sampling across Aeridinae to (1) illustrate the generic circumscription problem of *Holcoglossum*, (2) understand the difficulty in accepting/rejecting paraphyletic groups in *Holcoglossum*, and (3) investigate the evolution of morphological characters within *Holcoglossum*.

## Results

### Genetic Distances and Phylogenetic Relationships in Subtribe Aeridinae

The mean genetic distance among *Holcoglossum* species is 0.010 (ITS), 0.012 (*matK*) and 0.014 (*trnL-F*). The genetic distances between *Ascocentrum himalaicum*, two monotypic genera (*Ascolabium* and *Penkimia*) and *Holcoglossum s.s.* are below 0.020 respectively (Table 1). *Penkimia*, *Ascolabium,* and *Ascocentrum himalaicum* are closer to *Holcoglossum* s.s. than to any other relatives (Table 1).

**Table 1:** Average pairwise sequence distances between *Holcoglossum* s.s. and related genera.

Average genetic distance	ITS	*matK*	*trnL-F*
*Holcoglossum* s.s.	0.010	0.012	0.014
*Penkimia nagalandensis* vs. *Holcoglossum* s.s.	0.010	0.013	0.027
*Ascocentrum himalaicum* vs. *Holcoglossum* s.s.	0.011	0.019	0.018
*Ascolabium pumilum* vs. *Holcoglossum* s.s.	0.011	0.018	0.013
*Holcoglossum* s.s. vs. *Aerides*	0.052	0.016	0.028
*Holcoglossum* s.s. vs. *Ascocentrum*	0.035	0.014	0.031
*Holcoglossum* s.s. vs. *Papilionanthe*	0.020	0.038	0.010
*Holcoglossum* s.s. vs. *Vanda*	0.035	0.010	0.041
*Ascocentrum himalaicum* vs. *Aerides*	0.045	0.023	0.027
*Ascocentrum himalaicum* vs. other *Ascocentrum*species	0.032	0.022	0.031
*Ascocentrum himalaicum* vs. *Papilionanthe*	0.015	0.045	0.009
*Ascocentrum himalaicum* vs. *Vanda*	0.032	0.018	0.040
*Penkimia nagalandensis* vs. *Aerides*	0.045	0.017	0.032
*Penkimia nagalandensis* vs. *Ascocentrum*	0.029	0.016	0.022
*Penkimia nagalandensis* vs. *Papilionanthe*	0.015	0.038	0.017
*Penkimia nagalandensis* vs. *Vanda*	0.029	0.018	0.031
*Ascolabium pumilum* vs. *Aerides*	0.054	0.022	0.036
*Ascolabium pumilum* vs. *Ascocentrum*	0.033	0.020	0.040
*Ascolabium pumilum* vs. *Papilionanthe*	0.020	0.049	0.017
*Ascolabium pumilum* vs. *Vanda*	0.038	0.016	0.050

Within Aeridinae, the ITS dataset for 138 taxa consisted of 854 characters of which 321 were parsimony informative. The Bayesian trees of each dataset were congruent with the MP trees, except for some weakly supported nodes. The interrelationships among most of the genera in Aeridinae were unresolved. *Holcoglossum* s.s. was not monophyletic: *Ascolabium, Penkimia* and *Ascocentrum himalaicum* were nested within different clades of *Holcoglossum* (Figure S1). Four out of the five sampled *Ascocentrum* species (except for *A. himalaicum*) were nested in the clade that included *Vanda*, *Neofinetia* and *Christensonia* (Figure S1).

### Phylogenetic Analyses within a Reduced *Holcoglossum* Dataset

The ITS dataset displayed 749 characters, 118 of which were parsimony informative. The combined matrix of plastid *matK*, *trnH*-*psbA* and *trnL*-*F* sequences indicates 4287 sites of which 258 are parsimony informative (Table S1). The monophyly of the clade including *Holcoglossum* s.s., *Ascolabium*, *Penkimia* and *Ascocentrum himalaicum* is moderately or weakly supported based on the ITS and the combined plastid datasets, respectively (results not shown).

**Figure 1:**
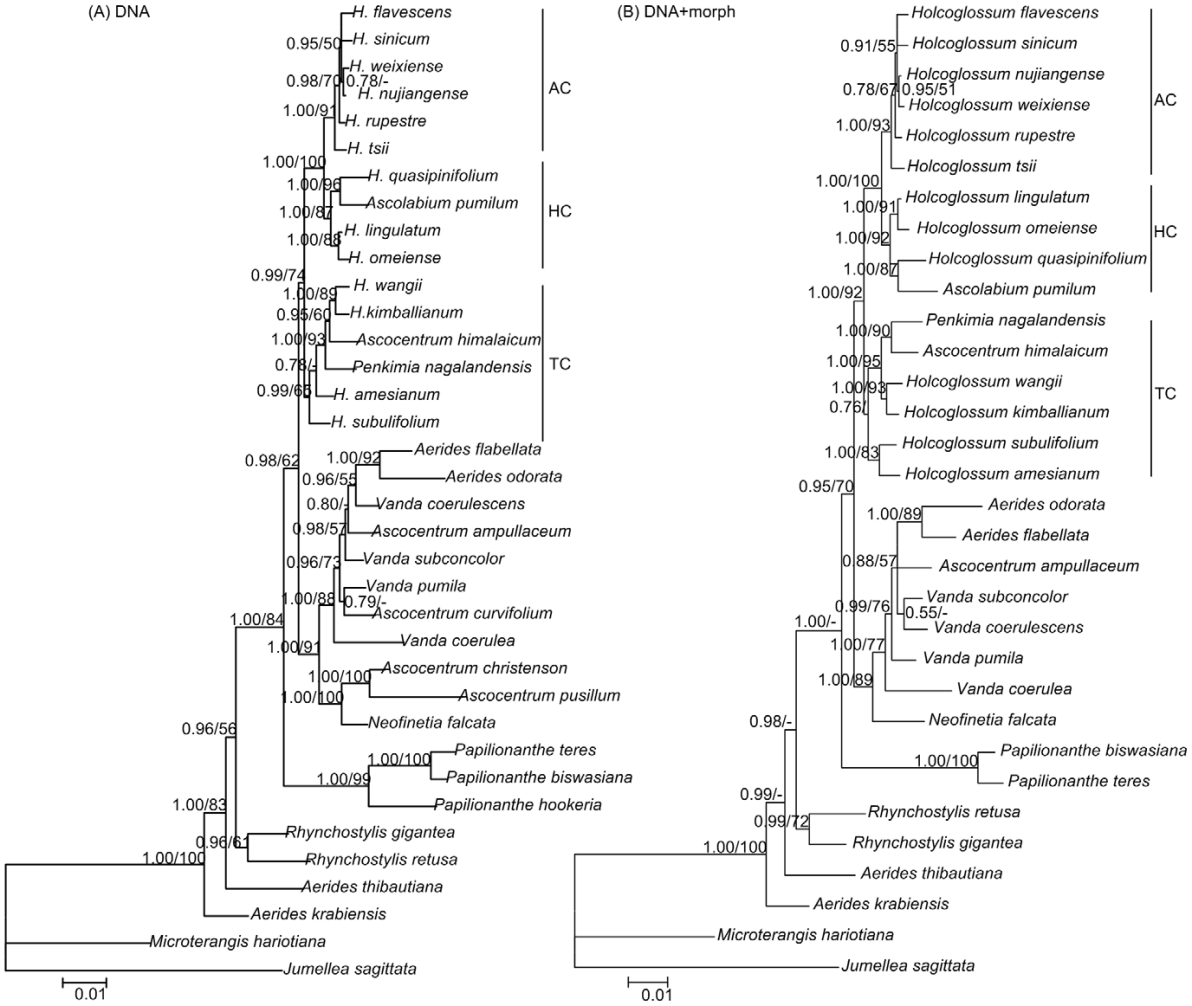
The 50% consensus Bayesian Inference and strict consensus maximum parsimony tree of *Holcoglossum* s.l. A. the combined DNA dataset, B. DNA and morphology dataset. The bootstrap percentages and posterior probability of >50% are shown above each branch.

The combined matrix of the nuclear and plastid datasets has 5036 sites, 376 of which are parsimony informative (Table S1). The Bayesian analysis yielded trees having topologies that are consistent with those obtained using MP analysis. The clade including *Holcoglossum* s.s., *Ascolabium*, *Penkimia* and *Ascocentrum himalaicum* is a monophyly with moderate support (BS = 74, PP = 0.99). Moreover, similar to the results of Fan et al. [32], this clade subdivided into three subclades, i.e., the tropical (TC), the alpine (AC) and the intermediate subclades (HC). *Penkimia* and *Ascocentrum himalaicum* are nested in TC (BS = 93, PP = 1.00), and *Ascolabium* is sister to *H. quasipinifolium* in HC (BS = 96, PP = 1.00) (Figure 1A).

In total, forty-five gross morphological characters are examined (Table S2). The cladograms from the morphological data based on the MP analysis were poorly resolved (Figure S2). Based on the combination of the morphological and molecular evidence, the clade comprising *Holcoglossum* s.s., *Ascolabium*, *Penkimia* and *Ascocentrum himalaicum* was strongly supported (BS = 92, PP = 1.00) (Figure 1B).

**Figure 2:**
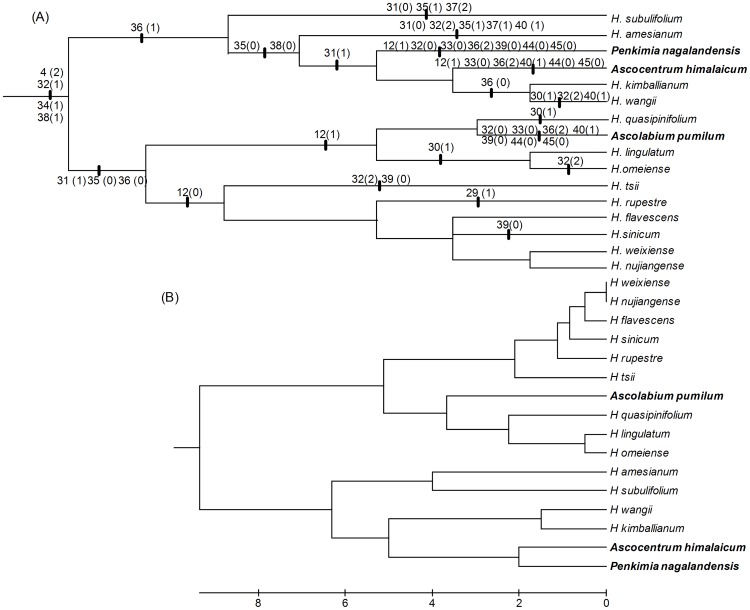
Dendrograms of relationships among *Holcoglossum*. A. Cladogram from Bayesian inference, B: Corresponding patrocladogram with equal weight.

The cladistic and patristic distances are shown in Table S3. The patrocladistic analysis revealed that *Ascolabium*, *Penkimia* and *Ascocentrum himalaicum* remain embedded within different subclades of *Holcoglossum* s.s. (Figure 2).

### Gross Morphology and Micromorphology

**Figure 3:**
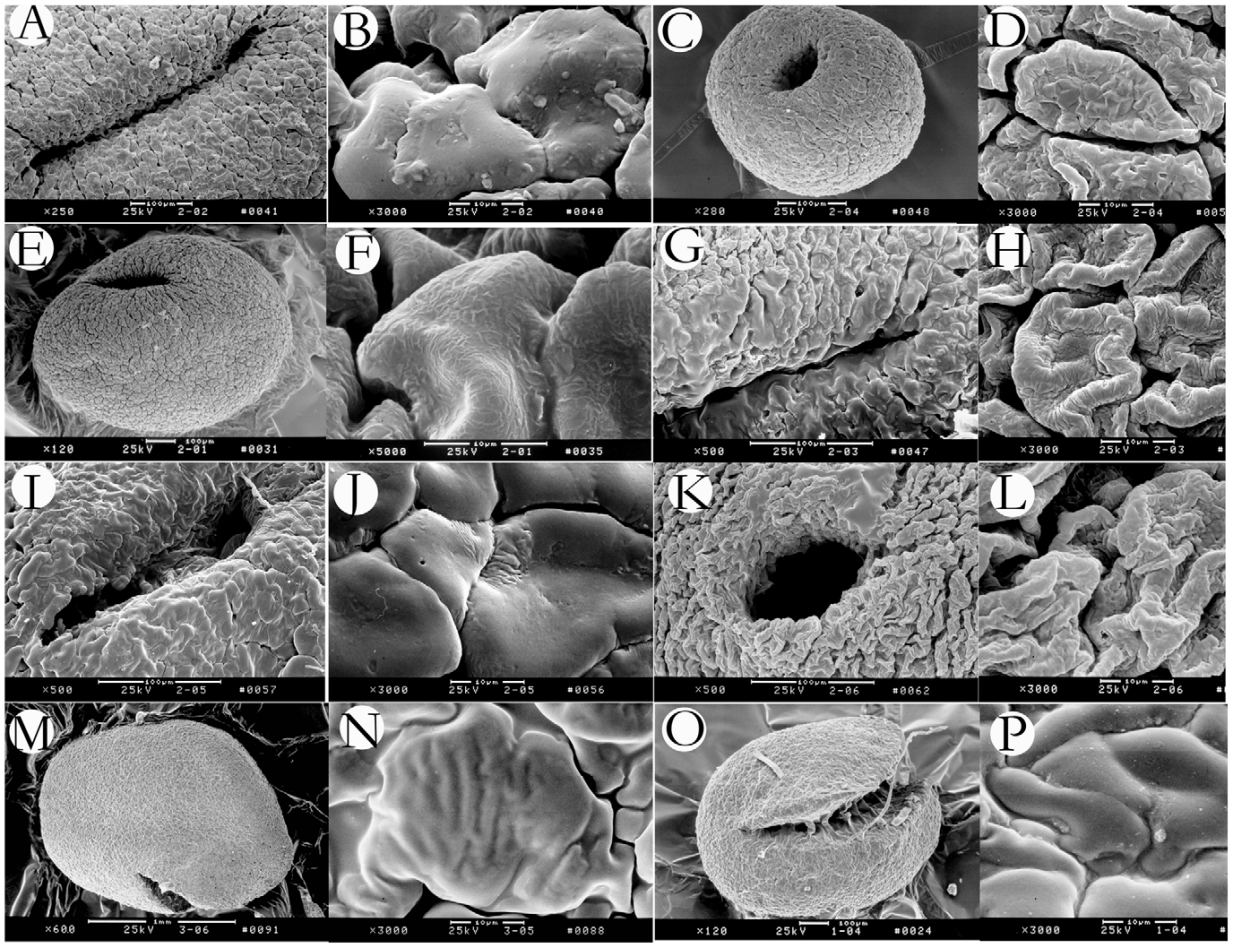
Pollinia of *Holcoglossum* and its alliance. A–B. *H. amesianum*; C–D. *H. himalaicum*; E–F. *H. kimabllianum*; G–H. *H. nujiangense*; I–J. *H. omeiense*; K–L. *H. rupestre*; M–N. *Vanda pumila*; O–P. *Aerides rosea*.

The pollinium micromorphology indicated that the pollinia of all eight examined species of *Holcoglossum* and *Ascocentrum* are porate, while the pollinia of *Vanda pumila* and *Aerides rosea* are uneven-split (Figure 3). The exine is psilate-scabrate in *Ascocentrum ampullaceum, Vanda pumila* and most species of *Holcoglossum*,and striato-reticulate in four alpine species: *H. sinicum, H. rupestre, H. nujiangense, H. weixiense* (Figure 3).

## Discussion

### Monophyly Versus Paraphyly − Definition of *Holcoglossum*

Based on the morphological and molecular evidence, our results indicated that *Holcoglossum* Schltr. (*Holcoglossum* s.s.) is paraphyletic; however, the clade including *Holcoglossum* s.s., *Ascolabium*, *Penkimia* and *Ascocentrum himalaicum* is strongly supported as a monophyly (Figure 1). The red to yellow tiny flowers misplaced members, namely, *Penkimia*, the neglected *Ascolabium* [34], and *Ascocentrum himalaicum*, have independently evolved at least twice in *Holcoglossum* s.s. (Figure S3). We are in a dilemma to circumscribe *Holcoglossum*. It appears that there are three proposals for the circumscription of *Holcoglossum*. (1) The first is to narrow *Holcoglossum*, as Liu et al. [33] did: based on this, at least six genera, *Holcoglossum, Ascolabium, Penkimia, Paraholcoglossum, Tsiorchis,* and another new genus containing *Ascocentrum himalaicum*, would be recognized, yet *Holcoglossum* would still be paraphyletic. (2) The second is to circumscribe *Holcoglossum* according to Jin [30] and Jin & Wood [35]: under this scenario, two monotypic or oligotypic genera, *Penkimia* (with *Ascocentrum himalaicum*) and *Ascolabium*, have to be recognized in addition to the paraphyletic *Holcoglossum* s.s. (3) The third is to define *Holcoglossum* sense lato: if *Holcoglossum* is redefined as monophyletic, then the other four genera, i.e., *Penkimia, Ascolabium, Paraholcoglossum,* and *Tsiorchis*, will be included, and *Ascocentrum himalaicum* will be transferred into it.

Regarding option 1, there are no distinct morphological characters to distinguish *Tsiorchis* and *Paraholcoglossum* from *Holcoglossum* (Figure S3), and the narrowly redefined *Holcoglossum* is still paraphyletic. Furthermore, the subtribe Aeridinae is abundant in many aberrant species and plagued by the many monotypic genera; therefore, this proposal would set a precedent to separate many aberrant species as new genera. Accordingly, it seems that the recognition of many similar monotypic genera while keeping *Holcoglossum* as paraphyletic provides no practicability or similarity and does not reflect the maximum evolutionary information.

With regard to option 2, all the members of the paraphyletic *Holcoglossum* s.s. are consistent in both the vegetative characters [22], [27], [30], [36] and floral characters, which epitomize bee-pollination syndrome [36], making it very good in practicability, high in similarity and information content; however, two small genera have to recognized, as their evolutionary information cannot be related to *Holcoglossum*.

**Figure 4:**
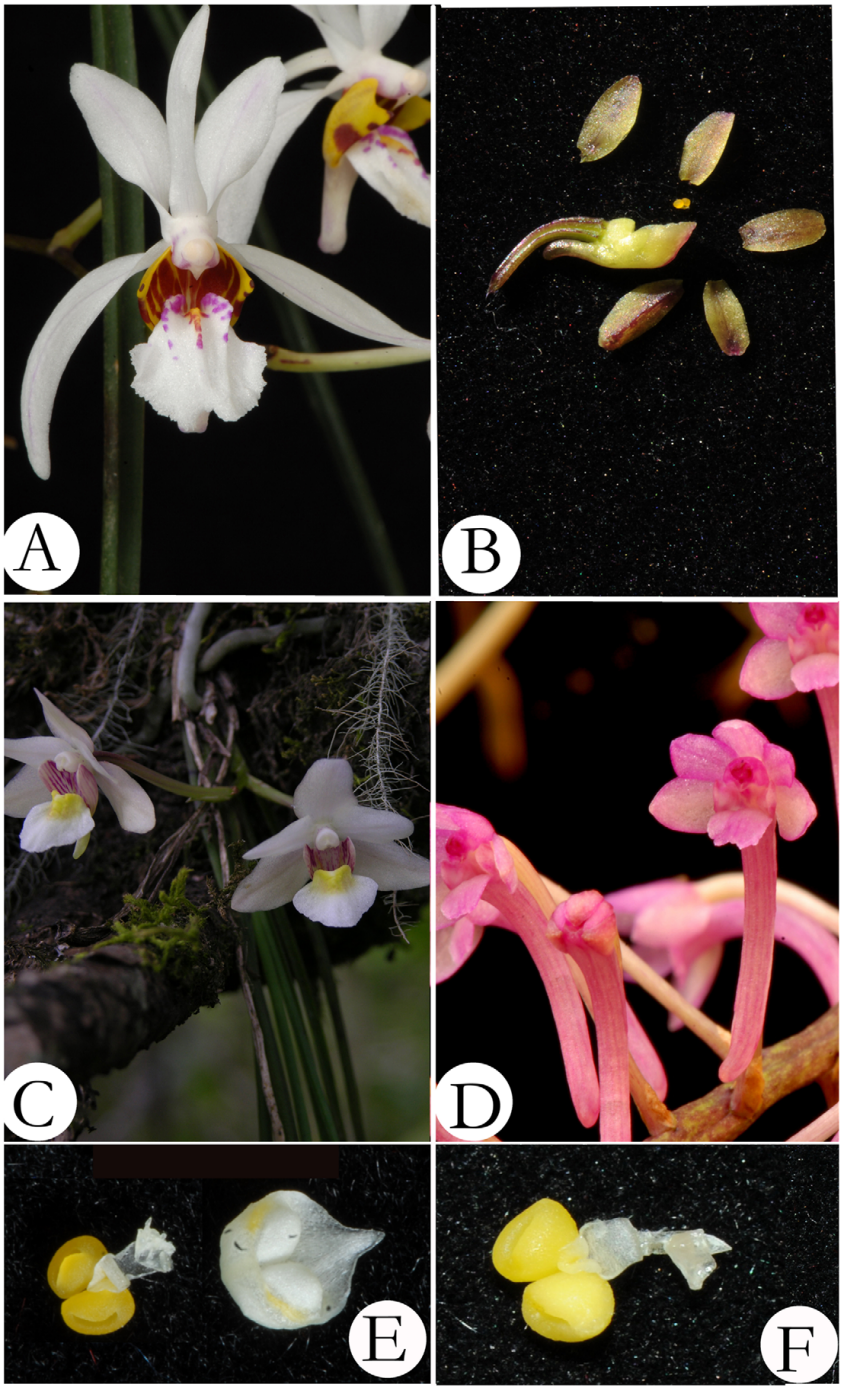
Flowers of *Holcoglossum*. A. *H. wangii*; B. *H. nagalandensis*; C. *H. nujiangense*; D. *H. himalaicum*; E. Pollinarium of *H. flavescens*; F. Pollinarium of *H. wangii*.

**Figure 5:**
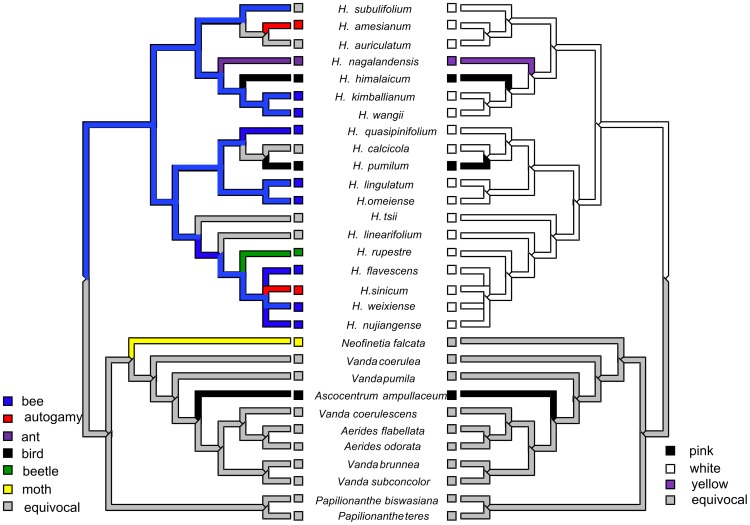
Relationships between pollination and flower color in *Holcoglossum*. The left panel shows diversity of the pollination types, and the right panel indicates the evolution of flower color.

For option 3, *Holcoglossum* s.l., including *Holcoglossum* s.s., two monotypic genera (*Ascolabium* and *Penkimia* ), *Ascocentrum himalaicum* and two newly described genera, *Paraholcoglossum* and *Tsiorchis*, is strongly supported as a monophyly by the molecular evidence and patrocladistic analyses (Figure 1 and 2). Indeed, the genetic distances revealed that these species show closer relationships to *Holcoglossum* s.s. than to other genera within Aeridinae (Table 1). In fact, *Ascocentrum himalaicum* was included in *Holcoglossum* by Tsi [28] and Averyanov [37] based on morphological characters. However, *Holcoglossum* s.l. is greatly heterogeneous in floral traits. The floral autapomorphies of *Ascolabium*, *Penkimia* and *Ascocentrum himalaicum*, such as red or purple, small-sized flowers, short and densely flowered inflorescences, and cylindrical spur, evolved independently at least twice in *Holcoglossum* and most likely were driven by the pollinator-mediated selection (Figure 4 and 5).

Based on these data, we tentatively propose to recognize a monophyletic circumscription of *Holcoglossum* that comprises approximately 17 species, including *Holcoglossum* s.s., *Ascolabium*, *Penkimia*, and *Ascocentrum himalaicum*, whereas *Paraholcoglossum* and *Tsiorchis* are placed within *Holcoglossum* s.s. for the purposes of practicability, similarity and providing the maximum evolutionary information.

### Morphological Character Evolution within *Holcoglossum* s.l

In comparison to the conserved vegetative characters, *Holcoglossum* s.l. is greatly divergent with regard to its floral characters (Table S2). Recent studies of pollination in *Holcoglossum* indicated that the pollination systems are more divergent than previously expected. To date, four pollination systems, autogamy [38], beetle pollination [39], bee pollination [40], and ant pollination [41], have been recorded in *Holcoglossum,* whereas bird pollination in *H. himalaicum* and *H. pumilum* remains to be confirmed [42]. Generally, pink flowers are considered bird pollination syndrome, and white flowers with a colored lip are considered bee pollination syndrome [43]. Two pink-flower species, *H. himalaicum* and *H. pumilum*, had previously been placed in *Ascocentrum* due to their floral similarities with *A. ampullaceum*, but our results indicated that pink flowers have independently evolved at least twice from white flowers in *Holcoglossum* s.l. (Figure 5, right). It appears that this shift is the result of pollinator-mediated selection (Figure 5, left). Therefore, *Holcoglossum* s.l. would be better to understand pollinator-driven floral divergence and vegetative stasis than a paraphyletic and narrowly defined genus.

### Conclusions

Based on the analyses of molecular and morphological characters, *Holcoglossum* s.s. is paraphyletic; the clade consisting of elements from five other genera, *Ascocentrum*, *Ascolabium*, *Paraholcoglossum*, *Penkimia* and *Tsiorchis*, is strongly supported as a monophyly. Hence, it would be better to retain *Holcoglossum* as monophyletic by the inclusiveness of three red to yellow species for the sake of information content, practicality and similarity. Therefore, we tentatively propose to recognize the monophyletic *Holcoglossum* s.l. (∼ 17 species).

### Taxonomic Treatment

***Holcoglossum*** Schltr., Repert Spec. Nov. Regni Veg. Beih. 4: 285. 1919;

Type. *Holcoglossum quasipinifolium* (Hayata) Schltr.

### Synonyms

1. ***Paraholcoglossum*** Z.J. Liu, S.C. Chen & L.J. Liu, PLoS One 6(10): e24864. 2011. syn. nov. **Type.** *Paraholcoglossum amesianum* (Rchb.f.) Z.J. Liu, S.C. Chen & L.J. Chen.
2. ***Tsiorchis*** Z.J. Liu, S.C. Chen & L.J. Liu, PloS One 6(10): e24864. 2011. syn. nov. **Type.** *Tsiorchis kimballianum* (Rchb.f.) Z.J. Liu, S.C. Chen & L.J. Chen.
3. ***Ascolabium*** S.S. Ying, Coloured Ill. Indig. Orchids Taiwan 1: 54. 1977. syn. nov. **Type.** *Ascolabium pumilum* (Hayata) S.S. Ying.
4. ***Penkimia*** Phukan & Odyuo, Orchid R*ev.* 114: 331. 2006. syn. no*v*. **Type.** *Penkimia nagalandensi* ***s*** Phukan & Odyuo.
5. ***Chenorchi***s Z. J. Liu, K.W. Liu et L. J. Chen, Acta Ecologica Sinica 28(6):2435. 2008. syn. nov. **Type.** *Chenorchis singchii* Z.J. Liu, K.W. Liu et L.J. Chen.

### Description

Epiphytic, small to moderate-size plants. Roots arising from the base of stem, white and fleshy, tip reddish. Stem short, usually clustered, enclosed by persistent leaf sheaths. Leaves articulate at base, condensed along stem, fleshy, subterete to terete, channeled adaxially. Inflorescences lateral. Flowers white to purple; pedicel and ovary long; dorsal sepal usually erect; lip 3-lobed, spurred or saccate; lateral lobes erect; middle lobe arising from spur; column winged; pollinia two, porate, usually with tapering stipe; anther cap beaked.

### Species

1. ***Holcoglossum amesianum*** (Rchb.f.) Christenson, Notes Roy. Bot. Gard. Edinburgh 44(2): 255. 1987. **Basionym.** *Vanda amesiana* Rchb.f. in Gard. Chron. 3 ser., 1: 764. 1887. **TYPE.** Myanmar (Burma). Shan States, comm. imp. Low anno 1887. Herb. Reichenbach 37196 (Holotype, W). **Homotypic synonym.** *Paraholcoglossum amensianum* (Rchb.f.) Z.J. Liu, S.C. Chen & L.J. Chen, PLoS One 6(10): e24864. 2011. syn. nov.
2. ***Holcoglossum calcicola*** Schuit. & P. Bonnet, Orchideen J. 16(1): 6. 2009. **TYPE.** Laos. Bolikhamxai Province, D. Barthélémy, P. Bonnet, A. Schuiteman, V. Lamxay PB 451 (Holotype, Herbarium of the Faculty of Sciences of the National University of Laos).
3. ***Holcoglossum flavescens.*** (Schltr.) Z.H. Tsi, Acta Phytotax. Sin. 20(4): 441. 1982. **Basionym.** *Aerides flavescens* Schltr., Fedde Repert. Sp. Nov. 19; 282. 1924. **TYPE.** China. Yunnan, Yunpe (Current Yongsheng), Simeon Ten 23 (Holotype, BD). **Homotypic** **synonyms.** *Papilionanthe flavescens* (Schltr.) Garay Bot. Mus. Leafl. Harvard Univ. 23(4):270. 1974. *Saccolabium yunpeense* T. Tang et F. T. Wang, Acta Phytotax. 1: 97. 1951.
4. ***Holcoglossum himalaicum.*** (Deb, Sengupta & Malick) Aver., Bot. Zhurn. (Moscow & Leningrad) 73: 432. 1988. **Basionym.** *Saccolabium himalaicum* Deb, Sengupta & Malick, Bull. Bot. Soc. Bengal 22: 213. 1968. **TYPE.** Myanmar. Sima, Shalik Mokin 13 (Holotype, CAL). **Heterotypic synonym.** *Holcoglossum junceum* Z.H. Tsi, Acta Phytotax. Sin. 20: 442.1982. **TYPE.** China. Yunnan, M.G. Li 1798 (Holotype, PE!); Z.H. Tsi 76 (Paratype, PE!).
5. ***Holcoglossum kimballianum.*** (Rchb.f.) Garay, Bot. Mus. Leafl. 23(4): 182. 1972. **Basionym.** *Vanda kimballiana* Rchb.f. in Gard. Chron. 3 ser., 5: 232. 1889. **TYPE.** Myanmar. Southern Shan States (comm. imp. s.n., Herb. Reichenbach 37216 (Holotype, W). **Homotypic synonyms.** *Tsiorchis kimballianum* (Rchb.f.) Z.J. Liu, S.C. Chen & L.J. Chen, PLoS One 6(10): e24864. 2011. syn. nov. **Heterotypic synonyms.** *Vanda saprophytica* Gagnep. in Bull. Soc. Bot. Fr. 79: 37. 1932. **TYPE.** Laos. Between Nong Het and Muang Seng, Tranninh 1400 m, Poliane 16918 (Holotype, P!). *H. saprophytica* (Gagnep.) Christenson in Not. Bot. Gard. Edinb. 44(2): 255. 1987.
6. ***Holcoglossum lingulatum.*** (Aver.) Aver., Consp. Sosud. Rast. Fl. Vietnama 1: 110. 1990. **Basionym.** *H. kimballianum* var. *lingulatum* Averyanov in Bot. J. (Leningr.) 73(3): 426. fig. 4. 1988. **TYPE.** Vietnam. Between Chapa and Hoan Lien Song, Takhtajan 0745 (Holotype, LE). **Heterotypic synonyms.** *H. tangii* Christenson in Lindleyana 13(2): 121–124. 1999. **TYPE.** China. Yunnan, without precise locality, Hort. Mountain Orchids s.n. (Holotype, K [spirit]!).
7. ***Holcoglossum nagalandensis.*** (Phukan & Odyuo) X.H. Jin, com. nov. **Basionym.** *Penkimia nagalandensis* Phukan & Odyuo, Orchid Rev. 114: 331. 2006. syn. nov. **TYPE**. India. Nagaland. Odyuo 102808A (Holotype, CAL).
8. ***Holcoglossum nujiangense*** X.H. Jin & S.C. Chen, Nordic J. Bot. 25(1–2): 127. 2008. **TYPE**. China. Yunnan, Fugong County, X.H. Jin 6930 (Holotype, PE!). **Heterotypic synonym.** *Holcoglossum linearifolium* Z.J. Liu, S.C. Chen & L. J. Chen, PLoS One 6(10): e24864. 2011. syn. nov. **TYPE**. China. Yunnan, Malipo, Z.J. Liu 4865 (Holotype, NOCC).
9. ***Holcoglossum omeiense.*** X.H. Jin & S.C. Chen, Kew Bull. 59(4): 633 (−635). 2005. **TYPE.** China. Sichuan, Mt. Omei, from Qing-yin Temple to Hongchun Ping, alt. 720–1000 m, 15 September 1963, K.H. Shing et K.Y. Lang 1365A (Holotype, PE!; Isotypes, PE!).
10. ***Holcoglossum pumilum.*** (Hayata) X.H. Jin, com. nov. **Basionym.** *Saccolabium pumilum* Hayata, Bot. Mag. (Tokyo) 20: 77. 1906. **TYPE**. China. Taiwan. Biōritsu, K. Fujii s.n.(Holotype, ?).
11. ***Holcoglossum quasipinifolium.*** (Hayata) Schltr. in Fedde. Repert. Sp. Nov. Beih. 4:285.1919. **Basionym.** *Saccolabium quasipinifolium Hayata*, Icon. Pl. Formos. 2: 144. 1912. **TYPE.** China. Taiwan, Nimandaira, Mt. Arisan, Hayata and Sasaki sine no. (Holotype, TI!; Isotype, TAI F!).
12. ***Holcoglossum rupestre*** (Hand.-Mazz.) Garay in Bot. Mus. Leafl. Harvard Univ. 23(4): 182. 1972. **Basionym.** *Vanda rupestris* Hand.-Mazz. in Anz. Akad. Wiss. Wiem. Math.-Nat. 62:241. 1925; Hand.-Mazz., Symb. Sin. 7: 1359. 1936. **TYPE.** China. Yunnan, Zhongdian (now Shang-ri-la), Hand.-Mazz. 8802 (Holotype, W; Isotypes, WU!, E!, K!).
13. ***Holcoglossum sinicum*** Christenson, Notes Roy. Bot. Gard. Edinburgh 44(2): 255. 1987. **TYPE.** China. Yunnan, Yangbi, SEBC 380 (Holotype, E; Isotypes, KUN!, AMES!).
14. ***Holcoglossum subulifolium*** (Rchb.f.) Christenson, Notes Roy. Bot. Gard. Edinburgh 44(2): 255. 1987. **Basionym.** *Vanda subulifolia* Rchb.f., Flora 69: 552. 1886. **TYPE**. Myanmar. Veitch comm. imp. Herb. Reichenbach 37215 (Holotype, W!). **Homotypic synonym.** *Paraholcoglossum subulifolium* (Rchb.f.) Z.J. Liu, S.C. Chen & L. J. Chen, PLoS One 6(10): e24864. 2011. syn. nov. **Heterotypic synonyms.** *Holcoglossum auriculatum* Z.J. Liu, S.C. Chen & X.H. Jin, J. Wuhan Bot. Res. 23(2): 154. 2005. **TYPE.** China. Yunnan, Malipo, Z.J. Liu 2758 (Herbarium, Shenzhen City Wutongshan Nursries). *Vanda watsonii* Rofle, Gard. Chron. 3.s. 37: 82, 123. fig. 52. 1906. **TYPE.** Vietnam. Annam, Micholitz s. n. (Holotype, K!).
15. ***Holcoglossum tsii*** T. Yukawa, Ann. Tsukuba Bot. Gard. 19: 1. 2000. **TYPE**. China. Yunnan, without precise locality, TNS 9512285 (Holotype, Hort. Tsukuba Botanical Garden!; Isotype, PE!).
16. ***Holcoglossum wangii*** Christenson, Lindleyana 13(2): 123. 1998. **TYPE.** China. Yunnan, Hort. Mountain Orchids s.n. (Holotype, K [spirit]!).
17. ***Holcoglossum weixiense*** X.H. Jin & S.C. Chen, Novon 14(2): 178 (−179; fig. 1). 2004. **TYPE.** China. Yunnan, Weixi, HK Kadoorie PT 3490 (Holotype, PE!).

## Materials and Methods

### Ethics Statement

The species collected here are not included in the checklist of Chinese Protected Species. The fieldwork was conducted under the permission of the authority of each natural reserve, specifically Gaoligongshan National Nature Reserve (Yunnan, China), Dali Cangshan-Erhai National Natural Reserve (Yunnan, China), Jianfengling National Nature Reserve (Hainan, China) and Wuzhishan National Nature Reserve (Hainan, China). No specific permits were required for the described field studies.

### Taxon Sampling

To determine the systematic positions of *Holcoglossum* and related genera, we sampled 78 genera and 138 samples, representing all of the major clades in the subtribe Aeridinae, based on previous molecular work (Table S4) [26], [44]. Secondly, we sampled a reduced matrix containing 36 species to analyze the intraspecific relationships within *Holcoglossum* (Table S5).

To facilitate consistency and convenience, *Paraholcoglossum* and *Tsiorchis* were referred to *Holcoglossum* throughout the Results.

### DNA Extraction, PCR and Sequencing

The total DNA was extracted from silica gel-dried materials using the modified CTAB method [45]. The ITS, *matK* and *trnH*-*psbA* primers used for the amplification and sequencing are listed in Xiang et al. [46], and the *trnL-F* primers are from Taberlet et al. [47]. The selected DNA regions were amplified using a standard polymerase chain reaction (PCR). The sequencing reactions were performed using the ABI Prism Bigdye Terminator Cycle Sequencing Kit (Applied Biosystems, ABI).

### Genetic Distance Analyses

To estimate the variation of the ITS, *matK* and *trnL*-*F* sequences across *Holcoglossum* and related genera, we calculated the pairwise genetic p-distance for each region using MEGA v. 4 [48]. These distances were initially used to evaluate the interspecific divergence with the Kimura 2-Parameter model (K2P). *TrnH*-*psbA* was excluded under this analysis because all of the sequences were from *Holcoglossum* and there was a paucity of related genera.

### Phylogenetic Analyses

Clustal X 1.83 [49] was used to obtain an initial alignment of the DNA sequences, followed by manual adjustment using BioEdit [50].

The phylogenetic analyses for each matrix were performed using the maximum parsimony (MP) and Bayesian inference (BI) methods in PAUP v4.0b10 [51] and MrBayes v3.0b4 [52], respectively.

For the MP analyses, heuristic searches were conducted with 1,000 replicates of random addition, with one tree held at each step during the stepwise addition, tree-bisection-reconnection (TBR) branch swapping, MulTrees in effect, and the steepest descent off. All of the characters were unordered and equally weighted, and the gaps were coded as missing data. To access the node support, bootstrap analyses [53] were performed using 1,000 replicates, with 10 random taxon additions and heuristic search options.

Prior to the Bayesian analysis, the Akaike Information Criterion (AIC) implemented in ModelTest version 3.7 [54], [55] was used to select the best-fit model of molecular evolution for each dataset. For the BI analyses, four chains of the Markov Chain Monte Carlo (MCMC) were run, sampling one tree every 1,000 generations for 5,000,000, starting with a random tree. Majority rule (>50%) consensus trees were constructed after removing the “burn-in period” samples (the first 20% of the sampled trees).

### Gross Morphology and Micromorphology

To understand the morphology of *Holcoglossum* and relative genera, we performed herbarium examination of specimens (Table S6) and fieldwork observations for gross morphology, and scanning electric microscope (using KYKY-1000B) for the pollinium micromorphology (Table S7). However, the morphological characters of following not Chinese native five species, *Aerides krabiensis, A. thibautiana*, *Holcoglossum calcicola*, *Jumellea sagittata*, *Microterangis hariotiana*, are from literature.

### Character Mapping

To identify the synapomorphies that are congruent with each of the major clades of *Holcoglossum* retrieved in the molecular tree and to assess the value of the characters used in the classifications, 45 morphological characters were selected. We used the exemplar method, scoring the morphological characters in the morphological matrix for the same species as used in the molecular analyses. The complete morphological matrix, coding 45 characters for the 35 taxa, is available in Table S2. We also prepared a combined morphological and molecular matrix that included the 32 taxa for character mapping.

Character evolution was reconstructed onto a 50% major consensus tree generated in PAUP using the parsimony ancestral state reconstruction in Mesquite v.2.75 [56]. All of the morphological characters were considered unordered and unweighted.

### Patrocladistic Analysis

We displayed the patrocladistic analysis according to Stussey and König [57]. This cladogram with high support based on Bayesian inference served as a structure for the patristic distance. The cladistic distances were calculated from the selected cladogram, and these values were placed in a new cladistic matrix. The patristic distance was defined as the number of apomorphic step changes separating two taxa on the cladogram. The patristic distance was then added to the cladistic distance to form the combined patrocladistic data matrix (Table S3). These combined distances are used as input into UPGMA (packages in MEGA) to construct the patrocladogram.

## Supporting Information

Figure S1 **Bayesian inference tree of subtribe Aeridinae based on ITS.** The bootstrap percentages and posterior probability of >50% are shown above each branch. “−” = no value. “*”represents data from Fan et al. [32], and “#”represents data from Liu et al. [33].
(JPG)

Figure S2 **The strict consensus maximum parsimony tree of** ***Holcoglossum*** **s.l. based on the morphological data.** The bootstrap percentages of >50% are shown above each branch.
(JPG)

Figure S3 **Character mapping of** ***Holcoglossum*** **s.l. and related genera.** See Table S2 for the character numbers and states.
(JPG)

Table S1 **Statistics from the phylogenetic analyses of the various datasets.**
(DOC)

Table S2 **Morphological data for the phylogenetic analyses.**
(DOC)

Table S3 **Matrix of cladistic (lower left) and patristic (upper right) distances among taxa.**
(DOC)

Table S4 **Taxa and GenBank accession numbers for the ITS sequences in phylogenetic analysis of subtribe Aeridinae.** A dash indicates missing data; *represent the sequences obtained in this study, and the remaining sequences are from GenBank.
(DOC)

Table S5 **Taxa and GenBank accession numbers for the ITS,** ***matK*** **,** ***trnL-F*** **and** ***trnH*** **-** ***psbA*** **sequences in phylogenetic analysis of** ***Holcoglossum*** **alliance.** A dash indicates missing data; *represent the sequences obtained in this study, and the remaining sequences are from GenBank.
(DOC)

Table S6 **Samples used in the gross morphology investigation.**
(DOC)

Table S7 **Samples used in the micromorphology.**
(DOC)
