# Supplementary figures and images for: Monophyly or Paraphyly– The Taxonomy of Holcoglossum (Aeridinae: Orchidaceae)
Source: PLoS One. 2012 Dec 14;7(12):e52050. doi: 10.1371/journal.pone.0052050 (PMC3522637; doi:10.1371/journal.pone.0052050)

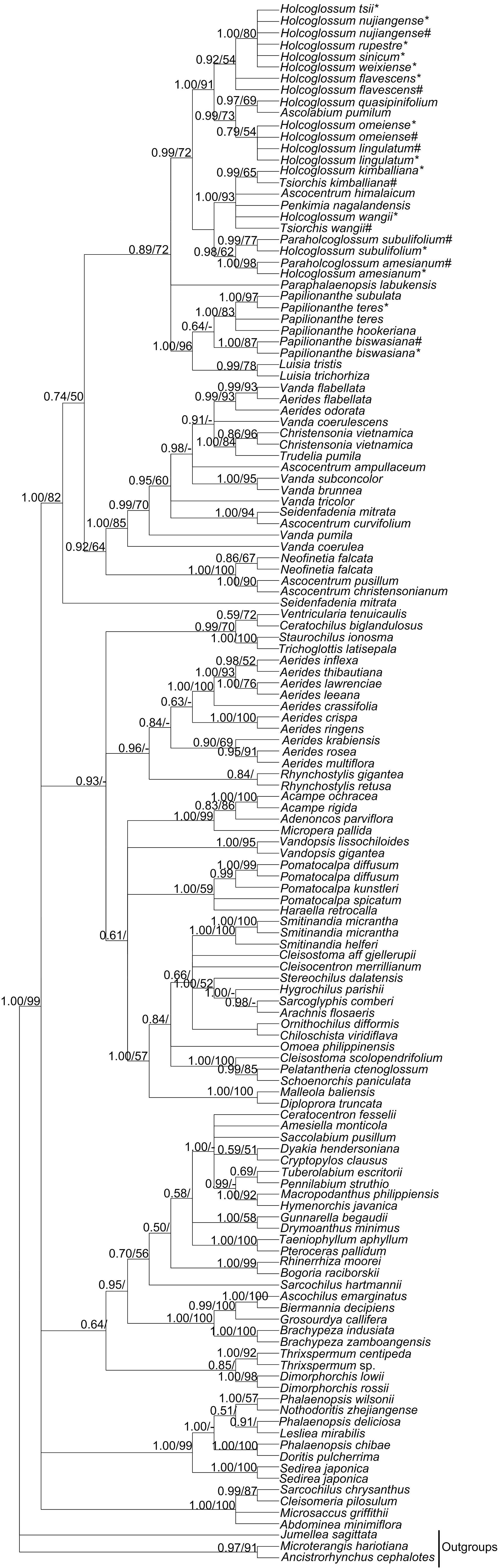

Supplement: Figure S1 — Bayesian inference tree of subtribe Aeridinae based on ITS. The bootstrap percentages and posterior probability of >50% are shown above each branch. “−” = no value. “*”represents data from Fan et al. [32], and “#”represents data from Liu et al. [33]. (JPG) [file pone.0052050.s001.jpg]

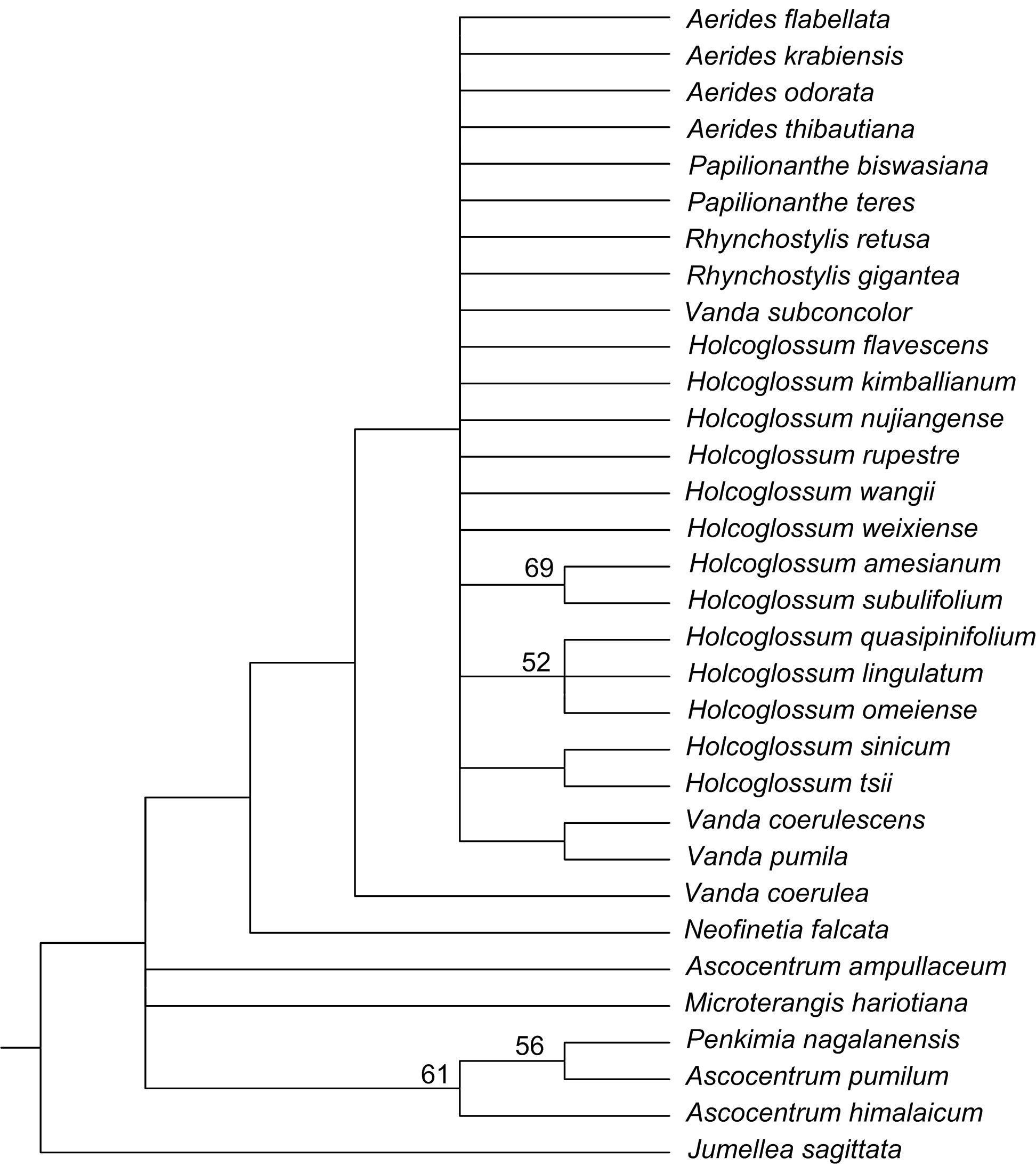

Supplement: Figure S2 — The strict consensus maximum parsimony tree of Holcoglossum s.l. based on the morphological data. The bootstrap percentages of >50% are shown above each branch. (JPG) [file pone.0052050.s002.jpg]

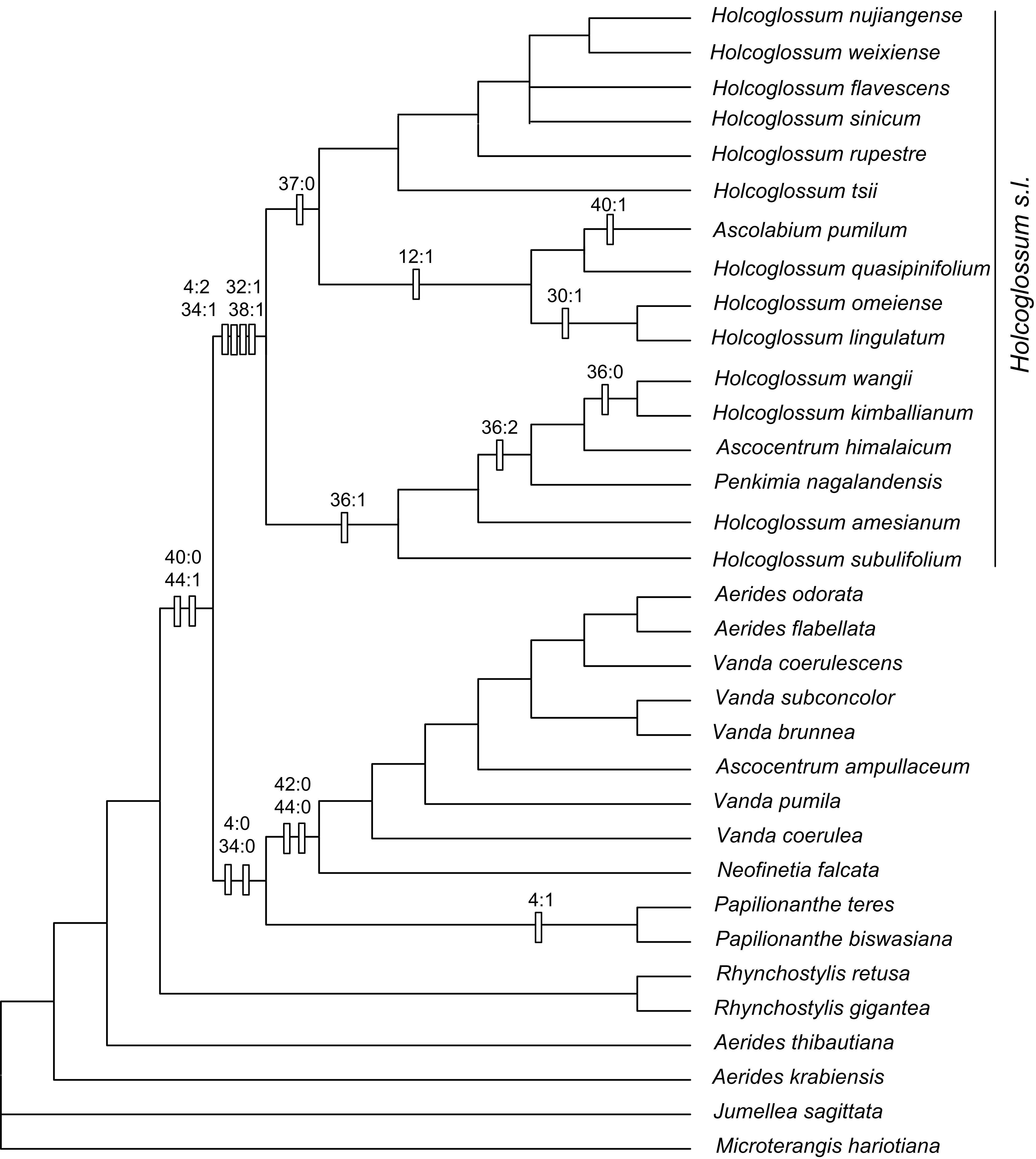

Supplement: Figure S3 — Character mapping of Holcoglossum s.l. and related genera. See Table S2 for the character numbers and states. (JPG) [file pone.0052050.s003.jpg]
