# Supplementary material for: Monophyly or Paraphyly– The Taxonomy of Holcoglossum (Aeridinae: Orchidaceae)
Source: PLoS One. 2012 Dec 14;7(12):e52050. doi: 10.1371/journal.pone.0052050 (PMC3522637; doi:10.1371/journal.pone.0052050)
Supplement: Table S1 — Statistics from the phylogenetic analyses of the various datasets. (DOC) [file pone.0052050.s004.doc]

Table S1 Statistics from the analyses of the various datasets

| Information | ITS | *matK* & *trnL*-*F*& *trnH*-*psbA* | Combined |
| --- | --- | --- | --- |
| No. of taxa | 36 | 36 | 36 |
| Aligned length | 749 | 4287 | 5036 |
| No. variable characters | 213 | 650 | 863 |
| No. informative characters | 118 | 258 | 376 |
| Tree length | 394 | 880 | 1292 |
| Consistency index (CI) | 0.675 | 0.820 | 0.765 |
| Retention index (RI) | 0.736 | 0.745 | 0.725 |
| Rescaled consistency index (RC) | 0.497 | 0.611 | 0.554 |
| Model | TrN+I+G | K81uf+I+G | K81uf+I+G |
