# Supplementary material for: Monophyly or Paraphyly– The Taxonomy of Holcoglossum (Aeridinae: Orchidaceae)
Source: PLoS One. 2012 Dec 14;7(12):e52050. doi: 10.1371/journal.pone.0052050 (PMC3522637; doi:10.1371/journal.pone.0052050)
Supplement: Table S2 — Morphological data for the phylogenetic analyses. (DOC) [file pone.0052050.s005.doc]

Table S2 Morphological data matrix for the phylogenetic analyses

| Taxa | Characters |
| --- | --- |
|  | 123456789012345678901234567890123456789012345 |
| *Holcoglossum amesianum* | 111211100010012100110110001200021111111101111 |
| *Holcoglossum flavescens* | 111211100010012100110110001200111100011001111 |
| *Holcoglossum kimballianum* | 111211100010012100110110001200111100001001111 |
| *Holcoglossum lingulatum* | 111211100011012100110110001201111100011001111 |
| *Holcoglossum nujiangense* | 111211100010012100110110001200111100011001111 |
| *Holcoglossum omeiense* | 111211100011012100110110001201121100011001111 |
| *Holcoglossum quasipinifolium* | 111211100011012100110110001201111100011001111 |
| *Holcoglossum rupestre* | 111211100010012100110110001210111100011001111 |
| *Holcoglossum sinicum* | 111211100010012100110110001200111100010001111 |
| *Holcoglossum subulifolium* | 111211100010012100110110001200011111211001111 |
| *Holcoglossum tsii* | 111211100010012100110110001200121100010001111 |
| *Holcoglossum wangii* | 111211100010012100110110001201121100001101111 |
| *Holcoglossum weixiense* | 111211100010012100110110001200111100011001111 |
| *Aerides flabellata* | 111011100000012100110110001200121001001101111 |
| *Aerides krabiensis* | 111011100010012100110110001200111000001100111 |
| *Aerides odorata* | 111011100010012100110110001200101000001100111 |
| *Aerides thibautiana* | 1110111000100121001101100012001?1000001100111 |
| *Ascocentrum ampullaceum* | 111011100010012100110110001200110100021100100 |
| *Ascocentrum himalaicum* | 111211100011012100110110001200110102001101100 |
| *Ascolabium pumilum* | 111211100011012100110110001200100102000101100 |
| *Neofinetia falcata* | 111011100010012100110110001200120000000000100 |
| *Papilionanthe biswasiana* | 111111100010012100110110001200121011101001111 |
| *Papilionanthe teres* | 111111100010012100110110001200101000101001111 |
| *Penkimia nagalanensis* | 111211100011012100110110001200100102000001100 |
| *Rhynchostylis retusa* | 111011100010012100110110001200101000101101111 |
| *Rhynchostylis gigantea* | 111011100010012100110110001200101000101101111 |
| *Vanda brunnea* | 111011100010012100110110001200121002101000111 |
| *Vanda coerulea* | 111011100010012100110110001200111000201000101 |
| *Vanda coerulescens* | 111011100010012100110110001200121000101100101 |
| *Vanda pumila* | 111011100010012100110110001200101002101100101 |
| *Vanda subconcolor* | 111011100010012100110110001200100000101100111 |
| Outgroups |  |
| *Jumellea sagittata* | 11101110001001210001011000120010111201110?100 |
| *Microterangis hariotiana* | 11101110001001210001011000120010020000110?000 |

**Morphological character:**

1. root epidermis 0=rhizodermis, 1=velamen

2. growth pattern 0=sympodial, 1=monopodial

3. phyllotaxy 0=spiral, 1=distichous

4. leaf morphology 0=flat, 1=terete, as wide as its own thick, 2=broadly subterete, 2-3times wider than thick

5. winter leaf 0=absent, 1=present

6. leaf articulation 0=absent, 1=present

7. inflorescence position 0=terminal, 1=lateral

8. calyculus 0=absent, 1=vanilloid, 2=polystachyoid

9. slipper-shaped labellum 0=absent, 1=present

10. apiculate sepals 0=absent, 1=present

11. carinate petals 0=present, 1=absent

12. lip-column marginal adnation 0=absent, 1=present

13. dorsal median stamen 0=present, 1=absent

14. lateral inner stamens 0=present, 1=absent

15. anther orientation 0=erect, 1=bending late, 2=bending early

16. operculate anther 0=absent, 1=present

17. basal caudicles 0=absent, 1=present

18. hammer stipe 0=absent, 1=present

19. tegula 0=absent, 1=present

20. pollen unit 0=monad, 1=tetrad

21. massulae 0=absent, 1=orchidoid, 2=epidendroid, 3=arethusoid

22. pollinium texture 0=granular, 1=solid

23. pollinium number= 2 0=absent, 1=present

24. pollinium number= 8 0=absent, 1=present

25. pollinium orientation 0=juxtaposed, 1=superposed

26. ovary locule number 0=one, 1=three

27. stigma 0=protruded, 1=sunken

28. viscidium 0=none, 1=diffuse, 2=detachable

29. Petal margins 0=entire or undulate, 1=denticulate

30. lip morphology 0=unlobed or trilobed with entire side-lobes, 1=trilobed with bilobed side-lobes

31. lip base 0=saccate, 1=spurred, 2=not saccate or spurred

32. lip appendages 0=absent, 1=present, in the middle of base of the middle lobe, 2=present, at the other part of the mid-lobe

33. column-foot 0=absent, 1=present

34. pollinium characters 0=cleft, 1=porate, 2=not cleft or porate

35. lip mid-lobe clawed at base 0=absent, 1=present

36. stipe 0=tapering, 1=oblong, 2=linear;

37. spur 0=cylindrical, 1=horn-shaped, 2=not cylindrical or horn-shaped

38. lip colour 0=purple or purple markings, 1=white, 2=not purple and purple markings or white

39. stem 0=short, 1=elongate

40. plant clustered 0=present, 1=absent

41. caudicle 0=present, 1=absent

42. reddish root tips 0=absent,1=present

43. column wing 0=absent, 1=present

44. uniformly color pattern in flowers 0=present, 1=absent,

45. lateral lobes not connecting with column 0=absent, 1= present
