# Supplementary material for: Monophyly or Paraphyly– The Taxonomy of Holcoglossum (Aeridinae: Orchidaceae)
Source: PLoS One. 2012 Dec 14;7(12):e52050. doi: 10.1371/journal.pone.0052050 (PMC3522637; doi:10.1371/journal.pone.0052050)
Supplement: Table S3 — Matrix of cladistic (lower left) and patristic (upper right) distances among taxa. (DOC) [file pone.0052050.s006.doc]

*Table S3 Matrix of cladistic (lower left) and patristic (upper right) distances among taxa.*

|  | 1 | 2 | 3 | 4 | 5 | 6 | 7 | 8 | 9 | 10 | 11 | 12 | 13 | 14 | 15 | 16 |
| --- | --- | --- | --- | --- | --- | --- | --- | --- | --- | --- | --- | --- | --- | --- | --- | --- |
| *H. flavescens* (1) |  | 1 | 0 | 0 | 1 | 2 | 3 | 9 | 3 | 4 | 12 | 9 | 14 | 15 | 12 | 8 |
| *H. sinicum* (2) | 0 |  | 1 | 1 | 2 | 3 | 4 | 10 | 4 | 5 | 13 | 10 | 15 | 16 | 13 | 9 |
| *H. weixiense* (3) | 1 | 1 |  | 0 | 1 | 2 | 3 | 9 | 3 | 4 | 12 | 9 | 14 | 15 | 12 | 8 |
| *H. nujiangense* (4) | 1 | 1 | 0 |  | 1 | 2 | 3 | 9 | 3 | 4 | 12 | 9 | 14 | 15 | 12 | 8 |
| *H. rupestre* (5) | 1 | 1 | 1 | 1 |  | 3 | 4 | 10 | 4 | 5 | 13 | 10 | 15 | 16 | 13 | 9 |
| *H. tsii* (6) | 2 | 2 | 2 | 2 | 1 |  | 5 | 11 | 5 | 6 | 14 | 11 | 16 | 17 | 14 | 10 |
| *H. quasipinifolium* (7) | 5 | 5 | 6 | 6 | 4 | 3 |  | 8 | 2 | 3 | 6 | 3 | 9 | 11 | 11 | 9 |
| *Ascolabium pumilum* (8) | 5 | 5 | 6 | 6 | 4 | 3 | 0 |  | 1 | 9 | 15 | 14 | 13 | 14 | 17 | 15 |
| *H. lingulatum* (9) | 5 | 5 | 6 | 6 | 4 | 3 | 2 | 2 |  | 1 | 10 | 7 | 12 | 13 | 12 | 9 |
| *H. omeiense* (10) | 5 | 5 | 6 | 6 | 4 | 3 | 2 | 2 | 0 |  | 11 | 8 | 13 | 13 | 12 | 10 |
| *H.* *wangii* (11) | 9 | 9 | 10 | 10 | 8 | 7 | 8 | 8 | 8 | 8 |  | 3 | 9 | 11 | 8 | 10 |
| *H. kimballianum* (12) | 9 | 9 | 10 | 10 | 8 | 7 | 8 | 8 | 8 | 8 | 0 |  | 6 | 8 | 5 | 7 |
| *Ascocentrum himalaicum* (13) | 8 | 8 | 9 | 9 | 7 | 6 | 7 | 7 | 7 | 7 | 1 | 1 |  | 3 | 11 | 12 |
| *Penkimia nagalandensis* (14) | 7 | 7 | 8 | 8 | 6 | 5 | 6 | 6 | 6 | 6 | 2 | 2 | 1 |  | 13 | 13 |
| *H. amesianum* (15) | 6 | 6 | 7 | 7 | 5 | 4 | 5 | 5 | 5 | 5 | 3 | 3 | 2 | 1 |  | 7 |
| *H.* *subulifolium* (16) | 5 | 5 | 6 | 6 | 4 | 3 | 4 | 4 | 4 | 4 | 4 | 4 | 3 | 2 | 1 |  |
