# Supplementary material for: Monophyly or Paraphyly– The Taxonomy of Holcoglossum (Aeridinae: Orchidaceae)
Source: PLoS One. 2012 Dec 14;7(12):e52050. doi: 10.1371/journal.pone.0052050 (PMC3522637; doi:10.1371/journal.pone.0052050)
Supplement: Table S4 — Taxa and GenBank accession numbers for the ITS sequences in phylogenetic analysis of subtribe Aeridinae. A dash indicates missing data; *represent the sequences obtained in this study, and the remaining sequences are from GenBank. (DOC) [file pone.0052050.s007.doc]

Table S4 Taxa and GenBank accession numbers for the ITS sequences in phylogenetic analysis of subtribe Aeridinae. A dash indicates missing data; * represent the sequences obtained in this study, and the remaining sequences are from GenBank.

| Taxon | Voucher | GenBank accession  no. ITS |
| --- | --- | --- |
| *Holcoglossum tsii* T.Yukawa |  | EU558927 |
| *Holcoglossum amesianum* (H.G. Reichenbach) Christenson |  | EU558906 |
| *Paraholcoglossum amesianum* (Rchb. f.) Z.J. Liu, S.C. Chen & L.J. Chen |  | JN106336 |
| *Holcoglossum flavescens* (Schltr.) Z.H. Tsi |  | EU558924 |
| *Holcoglossum flavescens* (Schltr.) Z.H. Tsi |  | HQ452904 |
| *Holcoglossum kimballianum* (Rchb.f.) Garay |  | EU558904 |
| *Tsiorchis kimballiana* (Rchb. f.) Z. J. Liu, S. C.  Chen & L. J. Chen |  | JN106331 |
| *Holcoglossum lingulatum* (Averyanov) Averyanov |  | EU558907 |
| *Holcoglossum lingulatum* (Averyanov) Averyanov |  | JN106334 |
| *Holcoglossum nujiangense* X.H. Jin & H. Li |  | EU558910 |
| *Holcoglossum nujiangense* X.H. Jin & H. Li |  | HQ452908 |
| *Holcoglossum omeiense* Z.H. Tsi ex X.H. Jin & S.C. Chen |  | EU558908 |
| *Holcoglossum omeiense* Z.H. Tsi ex X.H. Jin & S.C. Chen |  | JN106332 |
| *Holcoglossum quasipinifolium* (Hayata) Schltr. |  | HQ404411 |
| *Holcoglossum quasipinifolium* (Hayata) Schltr. |  | HQ452909 |
| *Holcoglossum rupestre* (Hand. -Mazz.) Garay |  | EU558920 |
| *Holcoglossum sinicum* Christenson |  | EU558919 |
| *Holcoglossum subulifolium* (Rchb.f.) Christenson |  | EU558905 |
| *Paraholcoglossum subululifolium* (Rchb. f.) Z.J. Liu, S.C. Chen & L.J. Chen |  | JN106335 |
| *Holcoglossum wangii* Christenson |  | EU558903 |
| *Tsiorchis wangii* (Christenson) Z.J. Liu, S.C. Chen & L.J. Chen |  | JN106333 |
| *Holcoglossum weixiense* X.H. Jin & S.C. Chen |  | EU558911 |
| *Abdominea minimiflora* J.J. Sm. |  | AB217524 |
| *Acampe ochracea* Hochr. |  | AB217525 |
| *Acampe rigida* (Buch. –Ham. Ex Sm.) P.F. Hunt |  | AB217526 |
| *Adenoncos parviflora* Ridl. |  | AB217527 |
| *Aerides crassifolia* C.S.P. Parish ex Burb. |  | EF670350 |
| *Aerides crispa* Lindl. |  | EF670351 |
| *Aerides flabellata* Rolfe ex Downie |  | AB217528 |
| *Aerides inflexa* Teijsm. & Binn. |  | EF670322 |
| *Aerides krabiensis* Seidenf. |  | EF670341 |
| *Aerides lawrenciae* Rchb.f. |  | EF670328 |
| *Aerides leeana* Rchb.f. |  | EF670324 |
| *Aerides multiflora* Roxb. |  | EF670349 |
| *Aerides odorata* Hour. |  | AB217529 |
| *Aerides ringens* (Lindl.) C.E.C. Fisch. |  | EF670353 |
| *Aerides rosea* Lind. & Paxt. |  | EF670340 |
| *Aerides thibautiana* Rchb.f. |  | EF670337 |
| *Ascocentrum ampullaceum* (Roxb.) Schltr. |  | AY912260 |
| *Ascocentrum christensonianum* J.R. Haager |  | AB217532 |
| *Ascocentrum curvifolium* (Lindl.) Schltr. Ex Prain |  | EF670356 |
| *Ascocentrum himalaicum* (Deb. Sengupta et Malick) Christenson | Jin X.H. 9496, Yunnan, China (PE) | KC110630* |
| *Ascolabium pumilum* (Hayata) Schltr. | Zhongsiwen, Taiwan, China | KC110631* |
| *Ascocentrum pusillum* Averyanov |  | AB217533 |
| *Amesiella monticola* J.E. Cootes & D.P. Banks |  | AB217530 |
| *Arachnis flosaeris* Rchb. f. |  | AB217531 |
| *Ascochilus emarginatus* (Blume) Schuit. |  | AB217534 |
| *Biermannia decipiens* (J.J. Sm.) Garay |  | AB217535 |
| *Bogoria raciborskii* J.J. Sm. |  | AB217536 |
| *Brachypeza indusiata* (Reichb.f.) Garay |  | AB217537 |
| *Brachypeza zamboangensis* (Ames) Garay |  | AB217538 |
| *Ceratocentron fesselii* Senghas |  | AB217539 |
| *Ceratochilus biglandulosus* Blume |  | AB217540 |
| *Cleisocentron merrillianum* (Ames) Christenson |  | AB217541 |
| *Cleisomeria pilosulum* (Gagnep.) Seidenf. & Garay |  | AB217542 |
| *Chiloschista viridiflava* Seidenf. |  | AB217543 |
| *Christensonia vietnamica* J.R. Haager |  | AB217544 |
| *Christensonia vietnamica* J.R. Haager |  | EF670357 |
| *Cleisostoma aff. g**jellerupii* (J.J. Sm.) Garay |  | AB217545 |
| *Cleisostoma scolopendrifolium* (Makino) Garay |  | AB217546 |
| *Cryptopylos clausus* (J.J. Sm.) Garay |  | AB217547 |
| *Dimorphorchis rossii var. graciliscapa* A.L.Lamb & Shim |  | EF670358 |
| *Dimorphorchis lowii* Rolfe |  | AB217548 |
| *Diploprora truncata* Rolfe ex Downie |  | AB217549 |
| *Doritis pulcherrima* Lindl. |  | AB217550 |
| *Drymoanthus minimus* (Schltr.) Garay |  | AB217551 |
| *Dyakia hendersoniana* (Rchb. f.) Christenson |  | AB217552 |
| *Grosourdya callifera* Seidenf. |  | AB217553 |
| *Gunnarella begaudii* (N. Halle) Senghas |  | AB217554 |
| *Haraella retrocalla* Kudo |  | AB217555 |
| *Hygrochilus parishii* Pfitzer |  | AB217557 |
| *Hymenorchis javanica* (Teijsmann & Bien.) Schltr. |  | AB217558 |
| *Lesliea mirabilis* Seidenf. |  | AB217559 |
| *Luisia trichorhiza* (Hook.) Blume |  | DQ091681 |
| *Luisia tristis* (G. Forest.) |  | EF670361 |
| *Macropodanthus philippiensis* Williams |  | AB217560 |
| *Malleola baliensis* J.J. Sm. |  | AB217561 |
| *Micropera pallida* Lindl. |  | AB217562 |
| *Microsaccus griffithii* (Par. & Rchb.f.) Seidenf. |  | AB217563 |
| *Neofinetia falcata* (Thunb.) Hu |  | AB217564 |
| *Neofinetia falcata* (Thunb.) Hu |  | AY912262 |
| *Nothodoritis zhejiangensis* Z.H. Tsi |  | AB217565 |
| *Omoea philippinensis* Ames |  | AB217566 |
| *Ornithochilus difformis* (Wall. Ex Lindl.) Schltr. |  | AB217567 |
| *Papilionanthe biswasiana* (Ghose et Mukerjee) Garay |  | EU558928 |
| *Papilionanthe biswasiana* (Ghose et Mukerjee) Garay |  | HQ452914 |
| *Papilionanthe teres* (Rohb.) Schltr |  | EU558934 |
| *Papilionanthe teres* (Rohb.) Schltr |  | FJ361771 |
| *Papilionanthe subulata* (Willd.) Garay |  | AB217568 |
| *Papilionanthe hookeriana* (Rchb. f.) Schlechter |  | FJ361770 |
| *Paraphalaenopsis labukensis* Shim, A.L. Lamb & C.L. Chan |  | EF670363 |
| *Pelatantheria ctenoglossum* Ridl. |  | AB217569 |
| *Penkimia nagalandensis* Phukan & Odyuo | Jin X.H. 8923, Yunnan, China (PE) | KC110629* |
| *Pennilabium struthio* Carr |  | AB217570 |
| *Phalaenopsis chibae* T. Yukawa |  | AB217572 |
| *Phalaenopsis deliciosa* Rchb.f. |  | AB217573 |
| *Phalaenopsis wilsonii* Rolfe |  | AB217575 |
| *Pomatocalpa diffusum* Breda |  | EF670364 |
| *Pomatocalpa diffusa* Breda |  | AB217576 |
| *Pomatocalpa kunstleri* J.J. Sm |  | AB217577 |
| *Pomatocalpa spicatum* Breda, Kuhl & Hasselt |  | EF670365 |
| *Pteroceras pallidum* (Bl.) Holttum |  | AB217578 |
| *Rhinerrhiza moorei* (Rchb.f.) M.A. Clem., B.J. Wallace & D.L. Jones |  | AB217579 |
| *Rhynchostylis gigantea* (Lindl.) Ridl. |  | AY912264 |
| *Rhynchostylis retusa* (L.) Blume |  | EU558933 |
| *Saccolabium pusillum* Bl. |  | AB217580 |
| *Sarcochilus hartmannii* F. Mueller |  | AB217581 |
| *Sarcochilus chrysanthus* Schltr. |  | AB217582 |
| *Sarcoglyphis comberi* (J.J. Wood) J.J. Wood |  | AB217583 |
| *Schoenorchis paniculata* Bl. |  | AB217584 |
| *Sedirea japonica* (L. Linden & Rchb.f.) Garay & H.R. Sweet |  | AB217585 |
| *Sedirea japonica* (L. Linden & Rchb. f.) Garay & H.R. Sweet |  | EF670378 |
| *Seidenfadenia mitrata* (Rchb. f.) Garay |  | AB217586 |
| *Seidenfadenia mitrata* (Rchb.f.) Garay |  | EF670379 |
| *Smitinandia helferi* (Hk. f.) Garay |  | AB217587 |
| *Smitinandia micrantha* (Lindl.) Holttum |  | AB217588 |
| *Smitinandia micrantha* (Lindl.) Holttum |  | EF670385 |
| *Staurochilus ionosma* Schltr. |  | AB217589 |
| *Stereochilus dalatensis* (Guillaumin) Garay |  | EF670386 |
| *Taeniophyllum aphyllum* Makino |  | AB217590 |
| *Thrixspermum sp.* |  | EF670367 |
| *Thrixspermum centipeda* Lour. |  | AB217591 |
| *Trichoglottis latisepala* Ames |  | AB217593 |
| *Trudelia pumila* (Hook.f.) Senghas |  | AB217594 |
| *Tuberolabium escritorii* (Ames) Garay |  | AB217595 |
| *Vanda coerulea* Griff. Ex Lindl. |  | AB217596 |
| *Vanda coerulescens* Griff. |  | EU558931 |
| *Vanda flabellata* (Rolfe ex Downie) Christenson |  | EF670368 |
| *Vanda pumila* Hook.f. |  | EU558930 |
| *Vanda subconcolor* Tang et Wang |  | EU558929 |
| *Vanda tricolor* Lindl. |  | EF670373 |
| *Vandopsis gigantea* (Lindl.) Pfitzer |  | EF670376 |
| *Vandopsis lissochiloides* (Gaud.) Pfitzer |  | EF670377 |
| *Ventricularia tenuicaulis* (Hk. F.) Garay |  | AB217598 |
| **Outgroups** |  |  |
| *Ancistrorhynchus cephalotes* (Rchb.f.) Summerh. |  | EF670388 |
| *Microterangis hariotiana* (Kraenzl.) Senghas |  | AB217523 |
| *Jumellea sagittata* H. Perrier |  | AB217522 |
