# Supplementary material for: Monophyly or Paraphyly– The Taxonomy of Holcoglossum (Aeridinae: Orchidaceae)
Source: PLoS One. 2012 Dec 14;7(12):e52050. doi: 10.1371/journal.pone.0052050 (PMC3522637; doi:10.1371/journal.pone.0052050)
Supplement: Table S5 — Taxa and GenBank accession numbers for the ITS, matK , trnL-F and trnH - psbA sequences in phylogenetic analysis of Holcoglossum alliance. A dash indicates missing data; *represent the sequences obtained in this study, and the remaining sequences are from GenBank. (DOC) [file pone.0052050.s008.doc]

Table S5 Taxa and GenBank accession numbers for the ITS, *matK*, *trnL*-*F* and *trnH*-*psbA* sequences in phylogenetic analysis of *Holcoglossum* alliance. A dash indicates missing data; * represent the sequences obtained in this study, and the remaining sequences are from GenBank.

| Taxon | Voucher | GenBank accession no. ITS/*matK*/*trnL*-*F*/*trnH*-*psbA* |
| --- | --- | --- |
| *Holcoglossum amesianum* (H.G. Reichenbach) Christenson |  | EU558906/EU558946/EU558879/HQ404438 |
| *Holcoglossum flavescens* (Schltr.) Z.H. Tsi |  | EU558924/EU558962/EU558898/HQ404443 |
| *Holcoglossum kimballianum* (Rchb.f.) Garay |  | EU558904/EU558944/EU558881/HQ404452 |
| *Holcoglossum lingulatum* (Averyanov) Averyanov |  | EU558907/EU558949/EU558883/HQ404455 |
| *Holcoglossum nujiangense* X.H. Jin & H. Li |  | EU558910/EU558959/EU558892/HQ404459 |
| *Holcoglossum omeiense* Z.H. Tsi ex X.H. Jin & S.C. Chen |  | EU558908/EU558950/EU558882/HQ404461 |
| *Holcoglossum quasipinifolium* (Hayata) Schltr. | JXH028, Taiwan, China (PE) | HQ104411/JF763797/KC110638/HQ404463 |
| *Holcoglossum rupestre* (Hand. -Mazz.) Garay |  | EU558920/EU558951/EU558885/HQ404467 |
| *Holcoglossum sinicum* Christenson |  | EU558919/EU558956/EU558890/HQ404474 |
| *Holcoglossum subulifolium* (Rchb.f.) Christenson |  | EU558905/EU558943/EU558880/HQ404478 |
| *Holcoglossum tsii* T.Yukawa |  | EU558927/AB217732/EU558902/- |
| *Holcoglossum wangii* Christenson |  | EU558903/EU558945/EU558878/HQ404482 |
| *Holcoglossum weixiense* X.H. Jin & S.C. Chen |  | EU558911/EU558957/EU558889/HQ404485 |
| *Aerides flabellata* Rolfe ex Downie |  | AB217528/AB217704/EU558870/- |
| *Aerides krabiensis* Seidenf. |  | EF670341/EF655784/EF670404/- |
| *Aerides odorata* Hour. |  | AB217529/AB217705/EF670389/- |
| *Aerides thibautiana* Rchb.f. |  | EF670337/EF655813/EF670398/- |
| *Ascocentrum ampullaceum* (Roxb.) Schltr. |  | AY912260/EU558935/DQ194985/ JF925118 |
| *Ascocentrum christensonianum* J.R. Haager |  | AB217532/AB217708/-/- |
| *Ascocentrum curvifolium* (Lindl.) Schltr. Ex Prain |  | EF670356/EF655789/EF670423/- |
| *Ascocentrum himalaicum* (Deb. Sengupta et Malick) Christenson | Jin X.H. 9496, Yunnan, China (PE) | KC110630*/KC110640*/KC110636*/KC110633* |
| *Ascolabium pumilum* (Hayata) Schltr. | Zhongsiwen, Taiwan, China | KC110631*/KC110641*/KC110637*/KC110634* |
| *Neofinetia falcata* (Thunb.) Hu |  | AY912262/EF655782/DQ091442/- |
| *Papilionanthe biswasiana* (Ghose et Mukerjee) Garay |  | EU558928/EU558936/EU558871/- |
| *Papilionanthe teres* (Rohb.) Schltr |  | EU558934/EU558937/EU558872/- |
| *Papilionanthe hookeriana* (Rchb. f.) Schlechter |  | FJ361770/FJ495167/-/- |
| *Penkimia nagalandensis* Phukan & Odyuo | Jin X.H. 8923, Yunnan, China (PE) | KC110629*/KC110639*/KC110635*/KC110632* |
| *Rhynchostylis gigantea* (Lindl.) Ridl. |  | AY912264/AY557202/DQ194989/- |
| *Rhynchostylis retusa* (L.) Blume |  | EU558933/EU558938/EU558873/- |
| *Stereochilus dalatensis* (Guillaumin) Garay |  | EF670386/EF655796/EF670420/- |
| *Vanda coerulea* Griff. Ex Lindl. |  | AB217596/AB217772/GU185937/- |
| *Vanda coerulescens* Griff. |  | EU558931/EU558940/EU558875/- |
| *Vanda pumila* Hook.f. |  | EU558930/EU558941/EU558876/- |
| *Vanda subconcolor* Tang et Wang |  | EU558929/EU558942/EU558877/- |
| Outgroup |  |  |
| *Microterangis hariotiana* (Kraenzl.) Senghas |  | AB217523/AB217699/DQ091467/- |
| *Jumellea sagittata* H. Perrier |  | AB217522/AB217698/DQ091555/- |
