# Supplementary material for: Monophyly or Paraphyly– The Taxonomy of Holcoglossum (Aeridinae: Orchidaceae)
Source: PLoS One. 2012 Dec 14;7(12):e52050. doi: 10.1371/journal.pone.0052050 (PMC3522637; doi:10.1371/journal.pone.0052050)
Supplement: Table S6 — Samples used in the gross morphology investigation. (DOC) [file pone.0052050.s009.doc]

Table S6 Samples used in the gross morphology investigation.

| **Taxon** | **Locality** | **Voucher (PE)** |
| --- | --- | --- |
| *Holcoglossum amesianum* | Yunnan | Wang C.W. 72304 |
| *H. himalaicum* | Yunnan | Jin X.H. 9496 |
| *H. kimballianum* | Yunnan | Mao P.Y. 7175 |
| *H. nagalandensis* | Yunnan | Jin X.H. 8923 |
| *H. nujiangense* | Yunnan | Jin X.H. 6981 |
| *H. omeiense* | Sichuan | Xiong J.H. et al 32833 |
| *H. rupestre* | Yunnan | HK Kadoorie PT (Jin X.H.) 3495 |
| *H. subulifolium* | Hainan | HK Kadoorie PT (Jin X.H.) 3085 |
| *Vanda pumila* | Yunnan | Wang C.W. 73476 |
